# A common variant rs2054564 in *ADAMTS17* is associated with susceptibility to lumbar spondylosis

**DOI:** 10.1038/s41598-023-32155-w

**Published:** 2023-03-25

**Authors:** Yuki Taniguchi, Toru Akune, Nao Nishida, Go Omori, Kim HA, Kazuko Ueno, Taku Saito, Takeshi Oichi, Asako Koike, Akihiko Mabuchi, Hiroyuki Oka, Shigeyuki Muraki, Yasushi Oshima, Hiroshi Kawaguchi, Kozo Nakamura, Katsushi Tokunaga, Sakae Tanaka, Noriko Yoshimura

**Affiliations:** 1 Department of Orthopedics, The University of Tokyo Hospital, Hongo 7-3-1, Bunkyo-Ku, Tokyo, 113-8655 Japan; 2 Surgical Center, The University of Tokyo Hospital, Tokyo, 113-8655 Japan; 3 Hospital, National Rehabilitation Center for Persons with Disabilities, Tokorozawa, Saitama 359-0042 Japan; 4 Genome Medical Science Project, National Center for Global Health and Medicine, Tokyo, 162-8655 Japan; 5 Department of Health and Sports, Faculty of Health and Science, Niigata University of Health and Welfare, Niigata, 950-3198 Japan; 6 Division of Rheumatology, Department of Internal Medicine, Hallym University Sacred Heart Hospital, Anyang, 18450 Korea; 7 Healthcare Business Division, Hitachi, Ltd., Tokyo, 105-6412 Japan; 8 Department of Human Genetics, Graduate School of Medicine, The University of Tokyo, Tokyo, 113-8654 Japan; 9 Department of Medical Research and Management for Musculoskeletal Pain, 22nd Century Medical & Research Center, Faculty of Medicine, The University of Tokyo, Tokyo, 113-8654 Japan; 10 Department of Preventive Medicine for Locomotive Organ Disorders, 22nd Century Medical and Research Center, The University of Tokyo, Tokyo, 113-8654 Japan; 11 Orthopaedics and Spine Department, Tokyo Neurological Center, Tokyo, 105-0001 Japan; 12 Department of Orthopedics, Towa Hospital, Tokyo, 120-0003 Japan

**Keywords:** Genetic association study, Disease genetics, Cartilage, Skeleton, Musculoskeletal system, Spine regulation and structure

## Abstract

The molecular pathophysiology underlying lumbar spondylosis development remains unclear. To identify genetic factors associated with lumbar spondylosis, we conducted a genome-wide association study using 83 severe lumbar spondylosis cases and 182 healthy controls and identified 65 candidate disease-associated single nucleotide polymorphisms (SNPs). Replication analysis in 510 case and 911 control subjects from five independent Japanese cohorts identified rs2054564, located in intron 7 of *ADAMTS17*, as a disease-associated SNP with a genome-wide significance threshold (P = 1.17 × 10^–11^, odds ratio = 1.92). This association was significant even after adjustment of age, sex, and body mass index (P = 7.52 × 10^–11^). A replication study in a Korean cohort, including 123 case and 319 control subjects, also verified the significant association of this SNP with severe lumbar spondylosis. Immunohistochemistry revealed that fibrillin-1 (FBN1) and ADAMTS17 were co-expressed in the annulus fibrosus of intervertebral discs (IVDs). ADAMTS17 overexpression in MG63 cells promoted extracellular microfibrils biogenesis, suggesting the potential role of ADAMTS17 in IVD function through interaction with fibrillin fibers. Finally, we provided evidence of FBN1 involvement in IVD function by showing that lumbar IVDs in patients with Marfan syndrome, caused by heterozygous *FBN1* gene mutation, were significantly more degenerated. We identified a common SNP variant, located in *ADAMTS17*, associated with susceptibility to lumbar spondylosis and demonstrated the potential role of the ADAMTS17-fibrillin network in IVDs in lumbar spondylosis development.

## Introduction

**Figure 1:**
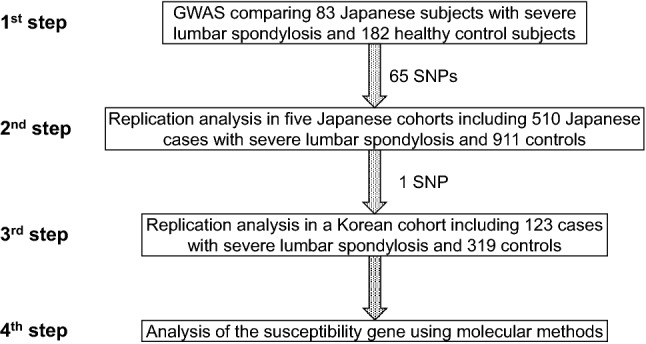
Summary flowchart of the present study.

Lumbar spondylosis, one of the most common skeletal diseases, is characterized by degeneration of intervertebral discs (IVDs) and osteophyte formation^1^. It is as prevalent as knee osteoarthritis (OA); however, lumbar spondylosis is less associated with job title and occupational activity than knee OA, indicating that genetic factors have a greater effect on the development of lumbar spondylosis^2,3^. Several genome-wide association studies (GWASs) or genetic association studies have been conducted on lumbar disc degeneration (LDD), which identified *ASPN, COL11A1, GDF5, SKT, THBS2, CILP*, *MMP9, CHST3,* and *PARK2* as susceptibility genes (Supplementary Table S1)^4–11^. However, as the pathophysiological relationship between LDD and severe lumbar spondylosis is not fully understood whether these susceptibility genes for LDD are also associated with lumbar spondylosis remains to be determined. Recently, two GWASs focusing on lumbar spondylosis have been conducted to overcome this gap, which identified susceptibility genes for lumbar spondylosis, including *BMP6*, *NIPAL1*, and *CNGA1* (Supplementary Table S1)^12,13^. However, because the number of identified susceptibility genes for lumbar spondylosis is still limited, little is known about the molecular pathophysiology underlying the development of lumbar spondylosis. Herein, to identify genetic factors associated with lumbar spondylosis, we conducted a GWAS and replication analysis using samples from Japanese individuals aged 50 years or older and identified one disease-associated single nucleotide polymorphism (SNP). A replication study in a Korean cohort verified the significant association of this SNP with severe lumbar spondylosis. Furthermore, to unveil the molecular pathogenesis of lumbar spondylosis, we investigated the function of the identified susceptibility gene in lumbar IVDs. A summary flowchart of the present study is depicted in Fig. 1.

## Results

### Screening for disease-associated SNPs using a GWAS

To examine genetic variants associated with severe lumbar spondylosis, we first conducted GWAS using 83 cases with severe lumbar spondylosis and 182 healthy controls. Case subjects with severe lumbar spondylosis were defined as those who had two or more levels of disc space narrowing (DSN) in the lumbar spine region. The Manhattan plot is shown in Supplementary Fig. S1. Among 502 SNPs with P-value < 1.0 × 10^–3^ identified using the allele frequency model, we selected 65 SNPs for further analysis based on the following: (1) For SNPs with P-value < 1.0 × 10^–4^, those with the smallest P-value from each linkage disequilibrium (LD) block was selected, (2) For SNPs with P-value < 1.0 × 10^–3^, we focused on those within gene regions and selected those with the smallest P-value from each LD block (Supplementary Table S2). Next, we conducted fine-mapping by inspecting the ± 500 kb region around each selected SNP using an imputed whole-genome sequence-based GWAS approach (Supplementary Table S2).

### Replication analysis in five independent cohorts

We analyzed the 65 SNPs using 510 cases and 911 controls from five independent Japanese cohorts, Tokyo-1, Tokyo-2, Wakayama-1, and Wakayama-2 from the ROAD project, and the Niigata cohort (Table 1)^14,15^*.* In this step, control subjects were defined as those without any mild DSN in the lumbar spine region. Since the Tokyo-1 cohort was used for first step screening, we first genotyped the 65 SNPs in all cases and controls of the remaining four cohorts: Tokyo-2, Wakayama-1, Wakayama-2, and Niigata, for replication analysis using a DigiTag2 assay^16,17^. Meta-analysis of these four cohorts using the Mantel–Haenszel test revealed that only one SNP, rs2054564, showed a significant association with lumbar spondylosis. (P = 3.05 × 10^–9^ < 0.00077 = 0.05/65) (Supplementary Table S2). Furthermore, meta-analysis of all five cohorts, including the Tokyo-1 cohort and four cohorts revealed that rs2054564 reached a genome-wide significance threshold (P = 1.17 × 10^–11^, odds ratio (OR) 1.92, 95% CI 1.60–2.32) (Table 2). The results of rs2054564 genotyping in each cohort are summarized in Table 2. Heterogeneity was not observed across the cohorts with respect to this SNP (I^2^ = 0). Since fine-mapping by imputed whole-genome sequence-based GWAS identified no other SNP with a smaller P-value than rs2054564 within the ± 500 kb region, this SNP was thought to be the disease-associated SNP (Supplementary Table S2). Taking into account the presence of rs2054564 in intron of the *ADAMTS17* (OMIM: 607511) gene, this gene was likely a susceptibility gene.

**Table 1:** Demographic data of each cohort.

Cohort	Ethnicity	Case (DSN ≥ 2)				Control			
		N	(M:F)	Age (years)	(SD)	N	(M:F)	Age (years)	(SD)
Tokyo-1	Japanese	94	(20:74)	78.7	(4.7)	153	(64:89)	76.5	(5.3)
Tokyo-2	Japanese	141	(29:112)	76.4	(5.2)	236	(98:138)	75.2	(6.0)
Wakayama-1	Japanese	105	(29:76)	72.3	(8.0)	206	(91:115)	72.0	(7.8)
Wakayama-2	Japanese	73	(25:48)	71.5	(8.8)	183	(73:110)	69.9	(7.0)
Niigata	Japanese	97	(28:69)	72.5	(7.6)	133	(63:70)	71.1	(7.6)
Total number of Japanese subjects		510	(131:379)	74.5	(7.3)	911	(389:522)	73.0	(7.2)
Korean	Korean	123	(47:76)	62.4	(5.6)	319	(158:161)	54.5	(7.7)

*DSN* disc space narrowing, *M* male, *F* female, *SD* standard deviation.

**Table 2:** Genotyping of rs2054564 in each cohort.

Cohort		Case (DSN ≥ 2)						Control						OR	95% CI	*P*-value
		N	CC	CT	TT	Freq of C	HWE *P*-value	N	CC	CT	TT	Freq of C	HWE *P*-value			
Tokyo-1	M	20	16	4	0	0.900	0.73	64	29	29	6	0.680	0.98	4.24	1.42–12.7	
	F	74	50	20	4	0.811	0.68	89	43	38	8	0.697	1.00	1.87	1.11–3.14	
Tokyo-2	M	29	16	11	2	0.741	0.86	98	50	39	9	0.709	0.96	1.18	0.61–2.28	
	F	112	71	36	5	0.795	0.95	138	61	64	13	0.674	0.92	1.87	1.24–2.82	
Wakayama-1	M	29	20	7	2	0.810	0.82	91	42	39	10	0.676	0.98	2.05	0.99–4.24	
	F	76	46	28	2	0.789	0.83	115	51	53	11	0.674	0.93	1.81	1.13–2.93	
Wakayama-2	M	25	15	10	0	0.800	0.53	73	35	32	6	0.699	0.98	1.73	0.79–3.76	
	F	48	31	15	2	0.802	0.86	110	53	37	20	0.650	0.14	2.18	1.23–3.87	
Niigata	M	28	19	7	2	0.804	0.82	63	25	34	4	0.667	0.46	2.05	0.96–4.36	
	F	69	45	20	4	0.797	0.68	70	27	37	6	0.650	0.65	2.12	1.23–3.63	
Meta-analysis of four cohorts (ex. Tokyo-1)		416	263	134	19	0.793	0.94	758	344	335	79	0.675	0.99	1.87	1.52–2.28	3.05E−09*
Meta-analysis of all cohorts		510	329	158	23	0.800	0.86	911	416	402	93	0.677	0.99	1.92	1.60–2.32	1.17 E−11*

*M* male, *F* female, *N* number; *Freq* frequency, *HWE* Hardy–Weinberg equilibrium, *OR* odds ratio, *CI* confidence interval, *DSN* disc space narrowing.

*Statistically significant with a genome-wide significance threshold of *P*-value < 5.0 × 10^–8^.

To determine the impact of the rs2054564 polymorphism on the development of severe lumbar spondylosis, we conducted logistic regression analysis to adjust for covariates, including age, sex, and body mass index (BMI). Even after adjustment, this SNP showed a significant association with severe lumbar spondylosis with a genome-wide significance threshold (P = 7.52 × 10^–11^, OR 1.88, 95% CI 1.55–2.27) (Table 3). Furthermore, when cases with severe lumbar spondylosis were stratified according to the severity of the spondylosis, the odds ratio increased in a dose-dependent manner in accordance with the number of DSN, albeit the P-values were worse for more severe cases (Table 3). This was probably due to the reduced effect size caused by the smaller number of cases with more severe spondylosis. This result convinced us of the critical role of the rs2054564 polymorphism in the development of severe lumbar spondylosis.

**Table 3:** Impact of rs2054564 on the development of severe lumbar spondylosis in Japanese and Korean cohorts.

	Japanese cohort					Korean cohort					Japanese + Korean cohort		
	N	Freq of C	OR	95% CI	*P*-value	N	Freq of C	OR	95% CI	*P*-value	OR	95% CI	*P*-value
Control	911	0.677	–	–	–	319	0.647	–	–	–	–	–	–
Case													
DSN ≥ 2	510	0.800	1.88	1.55–2.27	7.52E−11*	123	0.699	1.37	0.96–1.96	0.086	1.71	1.45–2.02	1.68E−10*
DSN ≥ 3	245	0.827	2.21	1.70–2.88	4.43E−09*	56	0.741	1.73	1.04–2.87	0.034**	2.06	1.63–2.59	1.16E−09*
DSN ≥ 4	77	0.851	2.70	1.70–4.28	2.47E−05	27	0.778	2.10	1.01–4.35	0.047**	2.44	1.66–3.59	5.11E−06

Adjusted for age, sex, and BMI by logistic regression model.

*N* number, *DSN* disc space narrowing, *Freq* frequency, *OR* odds ratio, *CI* confidence interval.

*Statistically significant with *P*-value < 5.0 × 10^–8^, a genome-wide significance threshold.

**Statistically significant with *P*-value < 0.05.

### Replication study in a Korean cohort

To investigate the association of this SNP with severe lumbar spondylosis in other populations, we performed a replication study using a Korean cohort, which comprised 442 subjects, including 319 controls and 123 cases (Tables 1, 3). Among 123 cases in a Korean cohort, 56 cases were with DSN ≥ 3 and 27 cases were with DSN ≥ 4 (Tables 1, 3). Logistic regression model adjusting age, sex, and BMI verified the significant association of more severe cases with DSN ≥ 3 (P = 0.034, OR 1.73, 95% CI 1.04–2.87) and ≥ 4 levels (P = 0.047, OR 2.10, 95% CI 1.01–4.35) (Table 3). Furthermore, when Japanese and Korean cohorts were analyzed together, the association reached a genome-wide significance threshold for severe spondylosis cases with DSN ≥ 2 (P = 1.68 × 10^–10^, OR 1.71, 95% CI 1.45–2.02) and ≥ 3 levels (P = 1.16 × 10^–9^, OR 2.06, 95% CI 1.63–2.50) (Table 3). A dose-dependent increase in odds ratio in accordance with the number of DSN was verified both in the Korean cohort alone and the combined analysis of Japanese and Korean cohorts (Table 3). These results suggested that the rs2054564 polymorphism was associated with the development of lumbar spondylosis in Japanese and Korean populations.

### Potential role of ADAMTS17 in IVD

**Figure 2:**
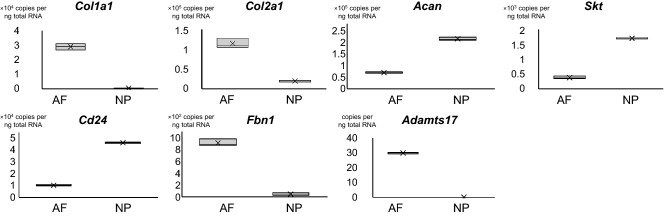
Expression levels of *ADAMTS17* and *FBN1* in the annulus fibrosus and nucleus pulposus in the mouse intervertebral disc. qRT-PCR revealed the expression of *Adamts17* and *Fbn1* in the annulus fibrosus (AF) of mouse intervertebral disc. High expression of *Col1a1* in AF, and high expression of *ACAN*, *Skt*, and *CD24* in nucleus pulposus (NP) confirmed the proper separated harvest of AF and NP tissues. Eight lumbar IVDs from two mice were collected to obtain enough tissues. All reactions were performed in triplicate.

**Figure 3:**
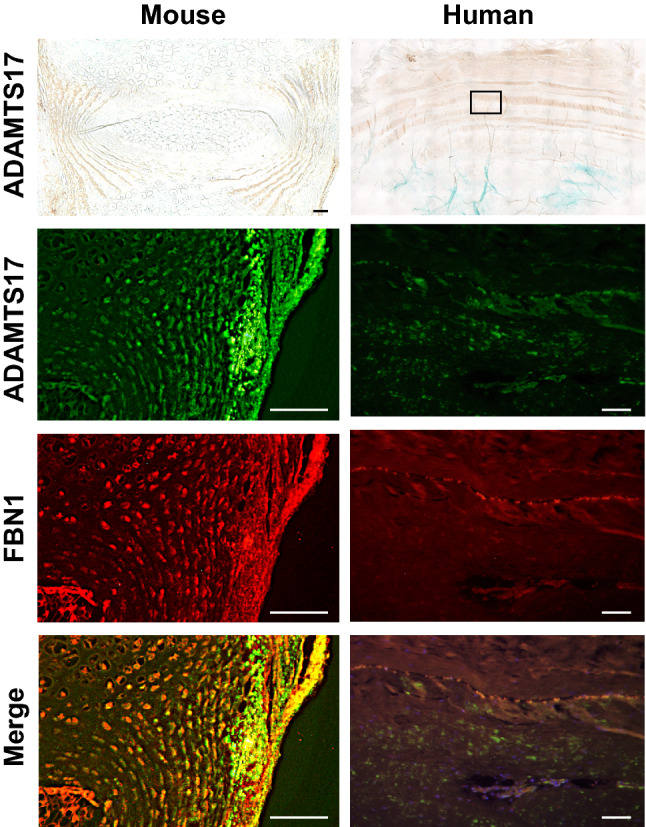
Immunohistochemistry and immunofluorescence images of ADAMTS17 and FBN1 in mouse and human intervertebral discs. Immunohistochemistry (the upper row) and immunofluorescence (the other rows) of ADAMTS17 and FBN1 in intervertebral disc (IVD) sections demonstrated the colocalized expression of Adamts17 and Fbn1 in annulus fibrosus of mouse (left) and human (right) IVDs. For mouse IVD sections, we used lumbar spinal IVDs obtained from C57/B6 mice of postnatal day 14 (P14). Non-degenerated human IVD samples were obtained from an 18-year-old patient during surgery for idiopathic scoliosis. Scale bar, 100 μm.

Since rs2054564 was located in intron 7 of the *ADAMTS17* gene and we identified this SNP by focusing on the lumbar DSN among several features of spondylosis, we investigated the potential role of ADAMTS17 in IVDs based on the hypotheses that this SNP may have an impact on ADAMTS17 expression or function, and ADAMTS17 may play a crucial role in IVDs. First, we conducted an expression analysis of ADAMTS17 in IVDs. qRT-PCR revealed that *Adamts17* was expressed in the annulus fibrosus (AF) but was hardly detected in the nucleus pulposus (NP) of a mouse IVD (Fig. 2). The expression levels of several genes in each tissue, including *Col1a1*, *Col2a1*, *Acan*, *Cd24*, and *Skt*, confirmed an adequately separated harvest of AF and NP tissues (Fig. 2)^18–20^. Expression profiles in AF and NP in the present study were consistent with those of the previous ones that reported high expression of *Col1a1* in AF and high expression of *ACAN*, *Skt*, and *CD24* in NP. Immunohistochemistry in both mouse and human IVD sections confirmed the localized expression of ADAMTS17 in AF, suggesting the potential role of ADAMTS17 within the AF of IVDs (Fig. 3).

**Figure 4:**
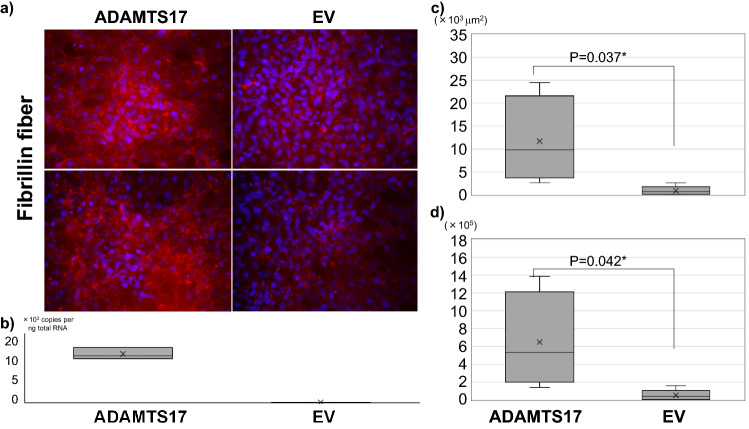
Promotion of extracellular microfibril biogenesis by ADAMTS17. (**a**) Immunocytochemistry of MG63 cells transfected with an expression vector of ADAMTS17 or empty vector (EV) revealed the promotion of extracellular microfibril assembly by exogenous expression of ADAMTS17. Red fibers represent extracellular fibrillin fibers. Nuclei are visualized using Hoechst 33342. Upper and lower rows show representative images of different experiments. (**b**) qRT-PCR confirmed the robust overexpression of ADAMTS17 in MG63 cells transfected with an expression vector of ADAMTS17. (**c**) Quantification of the extracellular microfibrils by positive areas confirmed significantly increased microfibril biogenesis by exogenous ADAMTS17 expression. (**d**) Quantification of the extracellular microfibrils by total signal intensities confirmed significantly increased microfibril biogenesis by exogenous ADAMTS17 expression. For quantification in both (**c,d**), the four images of ADAMTS17 group and the five images of EV group were automatically quantified using a hybrid cell count application in the BZ-X Analyzer software (KEYENCE, Japan). *EV* empty vector.

**Figure 5:**
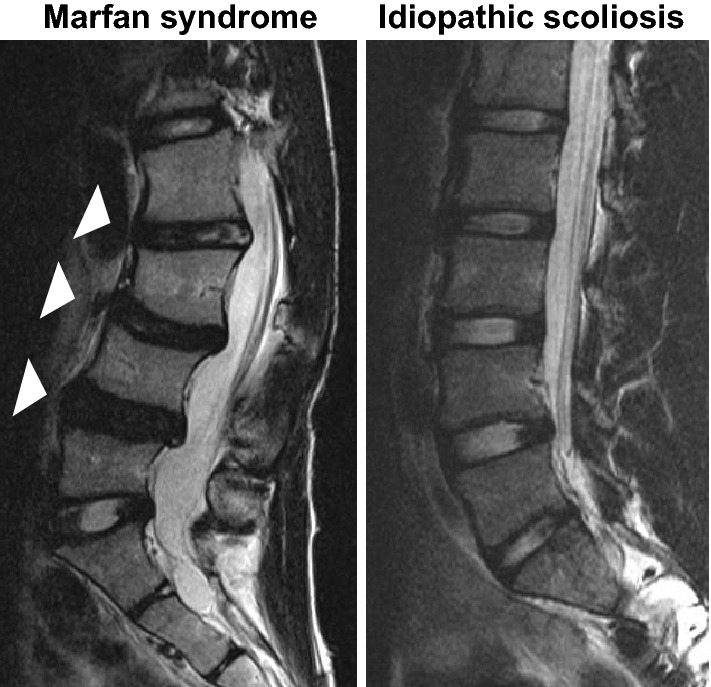
Representative preoperative magnetic resonance imaging (MRI) of the lumbar spine in patients with Marfan syndrome and idiopathic scoliosis. Representative preoperative MRI of the lumbar spine in patients with Marfan syndrome (left) and idiopathic scoliosis (right). Both cases were 14-year-old boys. White arrowheads indicate the degenerated intervertebral discs.

Although the target substrate of ADAMTS17 is not yet clearly defined, as mutations in *ADAMTS10* (OMIM: 608990), *ADAMTS17*, and *FBN1* (OMIM: 134797) can all lead to Weill-Marchesani syndrome (WMS) and ADAMTS10 promotes microfibril biogenesis through direct interaction with fibrillin-1, we hypothesized that ADAMTS17 promotes microfibril biogenesis through interaction with fibrillin fibers^21–27^. Hence, we next investigated the relationship between FBN1 and ADAMTS17. qRT-PCR verified the expression of *FBN1* in the AF, as previously reported (Fig. 2)^28–30^. Immunofluorescence confirmed the colocalization of ADAMTS17 and FBN1 in the AF of mouse and human IVDs, suggesting an interaction between these proteins (Fig. 3). Next, we examined the effects of exogenous expression of ADAMTS17 proteins on fibrillin-1 matrix assembly in MG63 cells, which have been shown to produce fibrillin-1-containing fibrillar matrices^31^. When we transfected MG63 cells with expression vectors encoding ADAMTS17 proteins, biogenesis of extracellular microfibrils was promoted (Fig. 4a). Overexpression of ADAMTS17 in MG63 cells was verified by qRT-PCR (Fig. 4b). Quantification of the extracellular microfibrils using both positive areas and signal intensities confirmed significantly increased microfibril biogenesis by exogenous ADAMTS17 expression (Fig. 4c,d). These results suggested that FBN1 is a potential substrate for ADAMTS17 and that ADAMTS17 plays essential roles in maintaining the homeostasis of the AF through interactions with fibrillin fibers. Finally, we investigated whether FBN1, the potential substrate of ADAMTS17, is involved in the IVD degeneration process. Since Marfan syndrome (OMIM: 154700) is caused by a heterozygous loss-of-function mutation of the *FBN1* gene, we analyzed lumbar IVDs in patients with Marfan syndrome^32,33^. Preoperative magnetic resonance images (MRIs) of the lumbar spine in patients with Marfan syndrome who underwent surgery for scoliosis in our hospital were compared with those in age- and sex-matched patients with idiopathic scoliosis. Lumbar IVDs of patients with Marfan syndrome were significantly more degenerated, which indicated the crucial role of FBN1 in maintaining the homeostasis of IVDs. (Table 4) Representative MRIs of the lumbar spine in patients with Marfan syndrome and idiopathic scoliosis of 14-year-old boys are shown in Fig. 5. Upper lumbar IVDs, which are less prone to degeneration than lower lumbar IVDs, were found to be significantly more degenerated in patients with Marfan syndrome (Table 4). These results suggest the critical role of FBN1 in IVDs and ADAMTS17 through interactions with FBN1 in IVDs. However, more investigation will be required to provide evidence on the possible interaction between ADAMTS17 and FBN1 within IVDs.

**Table 4:** Lumbar intervertebral disc degeneration in patients with Marfan syndrome and idiopathic scoliosis.

	Marfan group	IS group	*P*-value
N (Male: Female)	19 (6: 13)	38 (12: 26)	1
Age [range]	14.5 [10–21]	14.7 [10–22]	0.82
Cobb angle of main curve (°) [SD]	63.5 [24.5]	55.6 [8.9]	0.19
Cobb angle of TL/L curve (°) [SD]	48.5 [27.1]	35.2 [10.7]	0.051
Total number of degenerated IVD (%)	17/95 (17.9)	5/190 (2.6)	< 0.0001
L1/2–L3/4 N (%)	11/57 (19.3)	1/114 (0.9)	< 0.0001
L4/5–L5/S N (%)	6/38 (15.8)	4/76 (5.2)	0.07

Continuous variables were compared using a *t*-test. Categorical variables were compared using a chi-square test. Statistical significance was set at *P* < 0*.*05.

*N* number, *IS* idiopathic scoliosis, *TL/L* thoracolumbar/lumbar curve, *IVD* intervertebral disc, *SD* standard deviation.

## Discussion

In this study, we identified a common SNP variant in the *ADAMTS17* gene associated with susceptibility to lumbar spondylosis and suggested a potential role of ADAMTS17 in IVD function. To date, no previous study has identified an association between rs2054564 and any other disease. The frequency of the risk allele C of rs2054564 was reported to be 0.55 overall, 0.42 in Africans, 0.68 in Americans, 0.65 in East Asians, 0.65 in Europeans, and 0.42 in South Asians^34^. In the present study, we succeeded in identifying a SNP that reached a genome-wide significance threshold for a common disease like lumbar spondylosis using a relatively small number of subjects. This was partially owing to our strategy following which we strictly distinguished lumbar spondylosis case subjects from control individuals by eliminating ambiguous cases with only one level of DSN in the lumbar spine region in exchange for the decrease in subjects (Table 1). As subjects with multi-level DSN were considered to have a stronger genetic predisposition for spondylosis than those with a single level DSN, our strategy seemed to be effective, especially for identifying the genetic factors underlying the pathogenesis of spondylosis. Furthermore, the dose-dependent increase in odds ratio in accordance with the number of DSN convinced us that this common SNP is actually involved in the genetic pathogenesis of lumbar spondylosis in Japanese and Korean populations at least. Further replication studies in other populations are needed to understand whether this common SNP has a universal effect on the development of lumbar spondylosis. In addition, given the multifactorial nature of lumbar spondylosis, it is unlikely that this variant explains a large fraction of the risk of this disease; thus, further study with a larger number of subjects is essential to identify additional disease-associated SNPs.

Homozygous mutations in the *ADAMTS17* gene are known to cause Weill-Marchesani syndrome-4 (WMS4, OMIM: 613195), which is characterized by microspherophakia, severe myopia, glaucoma, cataract, brachydactyly, joint stiffness, and short stature^22–24^. A common SNP variant in ADAMTS17 has also been identified in a GWAS for short stature^35^. These previous reports led us to hypothesize that ADAMTS17 is involved in the skeletal development process, including spinal formation which consists of vertebral bone and IVD. Since these spinal components are all derived from the somitogenesis process, it is not surprising that ADAMTS17 may be related to the properties of IVDs.

ADAMTS17 belongs to the ADAMTS family of secreted proteases, which also comprises the ADAMTS-like proteins lacking proteolytic activity. ADAMTS proteases play pivotal roles in extracellular matrix (ECM) formation and homeostasis as well as in the pathological remodeling of the ECM by cleaving a variety of ECM substrates, such as procollagens, aggrecan, versican, fibronectin, and fibrillin-1 and -2^26,31,36–40^. The target substrate of ADAMTS17 is not yet clearly defined. Although fibrillin microfibril formation was not affected in vivo when analyzing the growth plate of *Adamts17−/−* mice, Karoulias showed altered fibrillin-1 deposition in vitro using skin fibroblasts derived from a patient with WMS4 caused by an *ADAMTS17* mutation and indicated a possible role of ADAMTS17 in the secretion of fibrillin-1 and its deposition in the ECM in human tissue^27,41^. These discrepancies may be partially explained by the differences in detection sensitivities between in vivo- and in vitro-assays or by the organ/species-specific role of each gene. ADAMTS17 is involved in the function of FBN1 in human tissues, and both heterozygous mutations of *FBN1* and homozygous mutations of *ADAMTS17* can cause WMS; these points strongly suggest the interaction between ADAMTS17 and FBN1, and we demonstrated that ADAMTS17 promotes extracellular microfibril biogenesis in vitro^22–25,27^*.* Further protein interaction assays, including co-immunoprecipitation or ligand affinity blotting, may elucidate the actual relationship between ADAMTS17 and FBN1. Regarding the expression distribution, ADAMTS17 has been reported to be widely expressed in many fetal and adult tissues in humans^22^. Previous in-situ hybridization studies in murine neonates demonstrated the robust expression of *Adamts17* in IVD, which is consistent with our results^42^. Hence, it is not surprising that ADAMTS17 was involved in the function of IVDs and a common SNP variant in the *ADAMTS17* gene was identified as a disease-associated SNP for lumbar IVD degeneration in the present study. Finally, we provided evidence that FBN1 is involved in normal IVD function by analyzing IVDs in patients with Marfan syndrome and suggested the potential role of ADAMTS17 in the AF of IVDs. Although several studies have demonstrated the expression of FBN1 in IVD, little is known about the function of FBN1 in IVD^28–30^. FBN1 is a key molecule of extracellular microfibrils and thus, can contribute to the mechanical properties of IVDs. Furthermore, fibrillin networks are known to modulate transforming growth factor β (TGFβ) signaling by regulating the bioavailability of TGFβ1^43,44^. Since dysregulation of TGFβ signaling has been repeatedly reported to be involved in the IVD degeneration process, it can be assumed that FBN1 plays crucial roles in IVD function^45,46^. Our study provided evidence for the involvement of FBN1 in the homeostasis of IVDs by demonstrating the fragility of IVDs in patients with Marfan syndrome. In the future, detailed analysis of IVDs in patients with WMS caused by *ADAMTS17* mutations may confirm the possible roles of ADAMTS17 in IVD function.

In the context of the earlier studies, previously identified disease-associated SNPs for LDD or lumbar spondylosis were not included in the 65 candidate SNPs selected from the first step GWAS of the present study^4–13^. This discrepancy was probably caused by our strict inclusion criteria for the lumbar spondylosis case subjects and control subjects, which might focus on a slightly different pathology from that of the previous studies. Conversely, it is interesting to note that *BMP6*, a previously identified susceptibility gene for lumbar spondylosis, and *ADAMTS17* had been both identified as susceptibility genes influencing adult human height in the same GWAS^13,35^. No report has investigated the direct molecular interaction or relationship between *BMP6* and *ADAMTS17*; however, this previous observation, though indirectly, suggests the possible functional link between these two genes on the development of lumbar spondylosis.

Although we investigated the functions of ADAMTS17 in IVDs on the assumption that the identified common SNP rs2054564, located in intron 7 of the *ADAMTS17* gene exerts effect on the expression of ADAMTS17, this hypothesis needs further discussion. Classically, intronic SNPs were supposed to target its located genes because introns are involved in regulating gene expression through splicing of mRNA or modulation of the mRNA translation. However, recent studies revealed that some noncoding variants might contribute to putative regulatory elements, and in that case, the target genes of intronic SNPs might be distal genes rather than its located genes. Hence, to elucidate the actual function of the identified common SNP rs2054564, further analysis is mandatory, which includes genotype–phenotype correlation analysis in human tissues or in vitro experiments using genome-edited human cells.

This study had some limitations. First, since the function of this common SNP was not analyzed in the present study, whether this SNP is actually involved in the function of ADAMTS17 remains unknown, as described above. Second, the function of ADAMTS17 in IVDs was not fully investigated. Third, because genetic association analysis was conducted only in Japanese and Korean subjects, whether this SNP possesses a universal effect in other populations remains to be elucidated. Fourth, the Korean cohort had a very small sample size, and the correlation in the replication study that used the Korean cohort was not very strong. This means that the results of the statistical analysis of the Korean cohort might be unstable.

## Conclusions

We identified a common SNP variant in the *ADAMTS17* gene that is associated with susceptibility to lumbar spondylosis and suggested a possible role of ADAMTS17 in IVD function through interactions with FBN1. To clarify the pathogenesis of lumbar spondylosis, further studies are necessary, including functional analyses of the SNP and replication analysis in other cohorts.

## Methods

### Study cohort

For the association analysis, we recruited subjects from five independent Japanese cohorts: Tokyo-1, Tokyo-2, Wakayama-1, Wakayama-2, and Niigata cohort. Among these five cohorts, the first four cohorts were prepared from the Japanese nationwide cohort study (Research on Osteoarthritis Against Disability; ROAD), and the Niigata cohort was prepared mainly from the Matsudai Knee Osteoarthritis Survey^2,14,15^. Details regarding the Korean cohort are available in published literature^12,47,48^. The Korean cohort was recruited from a rural farming community (Ansung). All subjects recruited from five independent Japanese cohorts were ≥ 50 years old or more. All subjects from the Korean cohort were ≥ 44 years old. The collection of specimens and clinical data from the different cohorts was approved by the Ethics Committee of the University of Tokyo (G1326-(6)). The study protocol for the Korean cohort was approved by the Ethics Committee of the Korean Health and Genome Study and Ajou University School of Medicine approved the study protocol (approval number AJIRB-CRO-07-012).

### Definition of the case and control subjects

Case subjects with severe lumbar spondylosis were defined as those with two or more levels of DSN in the lumbar spine region with DSN grade 2+, as reported in Lane et al.^49^. The control subjects in the first step (GWAS) comprised 184 healthy individuals residing in Tokyo, and no clinical information is available for these individuals^50^. The control subjects in the second step (replication analysis) were defined as those without mild DSN from L1/2 to L5/S IVDs on plain radiographs. The case subjects used for GWAS were recruited from a Tokyo-1 cohort. The remaining four cohorts, namely Tokyo-2, Wakayama-1, Wakayama-2, and Niigata cohorts were used for replication analysis^2,14,15^. Consequently, among 3,478 Japanese subjects from five independent cohorts, 510 case subjects with severe lumbar spondylosis and 911 control subjects were identified, and the remaining 2057 subjects with only one level of DSN in the lumbar spine were excluded from the analysis (Table 1). Informed consent was obtained from all participants.

### Genomic DNA samples and radiographs

One microgram of purified genomic DNA extracted from peripheral blood was resuspended in 100 mL of TE buffer (pH 8.0) (Wako, Japan), followed by storage at − 20 °C until use. Methods for extracting and storing genomic DNA from the Korean cohort have been described in detail in published papers^12,47^. Lateral radiographs of the lumbar spine were used for the evaluation of lumbar spondylosis. All lateral lumbar radiographs were obtained in the lateral decubitus position. The evaluation of the lumbar radiographs was conducted by a single experienced orthopedic surgeon (T.A.) in a blinded manner.

### SNP genotyping and data cleaning

For GWAS, we genotyped a total of 278 individuals, including 94 cases with severe lumbar spondylosis and 184 healthy Japanese individuals as controls, using the Affymetrix Genome-Wide Human SNP Array 6.0 for 900 K SNPs (Thermo Fisher Scientific, Inc., Waltham, MA, USA) according to the manufacturer’s instructions. The genotype calls of over 900 K SNPs were determined using Genotyping Console v4.1 software (Birdseed v1 algorithm) (http://tools.thermofisher.com/content/sfs/manuals/gtc_4_1_user_manual.pdf) which was provided by the manufacturer. Subjects with quality control call rate under 0.95 were excluded. Principal component analysis was conducted to check the genetic background in the studied samples together with the HapMap samples (Supplementary Fig. S2). In total, 265 samples consisting of 83 cases and 182 controls passed through these data cleaning steps and were used for subsequent statistical analysis.

The following thresholds for SNP quality control were applied: SNP call rate ≥ 0.95, minor allele frequency (MAF) ≥ 0.10, and Hardy–Weinberg equilibrium (HWE) P-value ≥ 0.001. Here, SNP call rate for each SNP is defined as the number of successfully genotyped samples divided by the number of total samples genotyped. A total of 462,470 SNPs on autosomal chromosomes cleared the quality control filters in the genome-wide association analysis.

### GWAS based on imputed genotypes

Prephasing was conducted using EAGLE version 2.4.1 with default settings (https://alkesgroup.broadinstitute.org/Eagle/)^51^. Genotype imputation was performed using IMPUTE4 with default settings (https://jmarchini.org/software/)^52^. The reference panel for genotype imputation was made in-house, comprising 10,176 haplotypes from 5088 individuals belonging to diverse populations, including 2493 individuals from the International 1000 Genomes, 820 individuals from the Human Genome Diversity Project, 241 individuals from the Simons Genome Diversity Project, 90 samples from the Korean Personal Genome Diversity Project, 1,026 Japanese individuals from Biobank Japan, and 418 Japanese individuals from Tokyo Healthy Control Project^50,53–56^. The Biobank Japan data are approved controlled access data from NBDC human data (JGAS000114), and the others were downloaded from public databases. Databases used for genotype imputation were summarized in (Supplementary Table S3). Imputed variants (SNPs and indels) with low quality (DR2 < 0.5) were filtered out, genotypes were hard called with the highest genotype probability, and genotype probabilities of less than 0.9 were considered no calls.

Association analysis was performed using PLINK version 1.9 (https://www.cog-genomics.org/plink/1.9/)^57^. The following parameters were used for PLINK: call rate > 97.0%, HWE P > 0.000001, MAF > 0.1%, and logistic regression modeling. Following this association analysis, five SNPs whose P-values were > 5.0 × 10^–8^ were removed as false positives as they did not have any other SNPs within 50 kb of the SNPs that have significant P-values. The final number of the associated SNPs was 4,995,213.

### Replication analysis using Japanese and Korean cohorts

The detail of the Japanese and Korean cohorts is provided in Table 1. A total of 65 SNPs with a P-value ≤ 0.001 in GWAS were selected for the replication analysis. SNP genotyping using 1421 Japanese samples, including 510 cases and 911 controls from five independent cohorts was completed for the selected 65 SNPs using the DigiTag2 assay or custom TaqMan PCR^16,17^. For the replication analysis in the Korean cohort, the target SNP was genotyped using 442 Korean samples, including 123 cases and 319 controls, with custom TaqMan PCR.

For meta-analysis of multiple cohorts for the selected 65 SNPs, we applied a fixed effect Mantel–Haenszel model with P-value < 0.00077 = 0.05/65 as the significance level. Each cohort was divided into two sub-cohorts according to sex, and all sub-cohorts were included in the meta-analysis independently. R program (v. 2.14.1) was used for the meta-analysis^58^. I^2^ statistic was calculated to test heterogeneity across the cohorts. Logistic regression models were used to test the SNPs additive effects on severe lumbar spondylosis after adjusting for covariates including age, sex, and BMI, using PLINK package (v1.07)^59^.

### Harvest of mouse IVD for qPCR

For qPCR, NP and AF of lumbar IVDs of the 8-week-old C57/B6 mice were harvested under a microscope. To obtain enough tissues for analysis, eight lumbar IVDs from two mice were collected. End plate tissues were eliminated carefully. The collected NP or AF tissues were directly processed using TRIzol Reagent (Invitrogen, USA), and total RNA was isolated from each tissue, using an RNeasy Mini Kit (Qiagen, Germany).

### Quantitative polymerase chain reaction (qPCR)

After isolation of total RNA, 1 μg of total RNA was reverse transcribed using a QuantiTect RT Kit (Qiagen, Germany) according to the manufacturer’s protocols. Quantitative PCR was performed with a Thermal Cycler Dice (Takara, Japan), using FastStart Universal SYBR Green Master (Roche, Switzerland). The mRNA copy number for each gene of interest was calculated using a standard curve generated from serially diluted plasmids containing PCR amplicon sequences. The copy number was normalized to mouse total RNA (Applied Biosystems, USA) with mouse β-actin or human total RNA (Applied Biosystems, USA) with human GAPDH as the internal control. All reactions were performed in triplicate. Primer sequences are shown in Supplementary Table S4.

### Expression plasmids and transfection

We amplified 3288 base pairs of protein-coding sequences of the *ADAMTS17* gene (reference sequence; NM_139057.4) from human complementary DNA (cDNA) and cloned them into a pcDNA3.1(+) vector (Invitrogen, USA). We verified that no mutations were introduced during PCR or cloning by Sanger sequencing. Transfection of vectors was conducted using lipofectamine 2000 (Invitrogen, USA).

### Human samples

We obtained samples of human IVD from an 18-year-old patient during surgery for idiopathic scoliosis. The harvested IVD was verified not to be degenerated on preoperative MRI. Written informed consent was obtained, and approval was provided by the Ethics Committee of the University of Tokyo (2674-(6)).

### Immunohistochemistry

For immunohistochemistry of mouse samples, we used C57/B6 mice of postnatal day 14 (P14). The lumbar spine was fixed in 4% paraformaldehyde for 24 h, decalcified in 10% EDTA at 4 °C for 3 weeks, embedded in paraffin wax, and sectioned at 5-mm thickness along the mid-sagittal plane. For immunohistochemistry of human IVD samples, the harvested IVD was fixed in 4% paraformaldehyde for 24 h, decalcified in 10% EDTA at 4 °C for 3 weeks, embedded in paraffin wax, and sectioned at 5-mm thickness along the axial plane. The sections were deparaffinized with xylene and incubated with 2.5% hyaluronidase (Sigma, St. Louis, MO) for 45 min at 37 °C. For immunohistochemistry, we used antibodies against Adamts17 (1:100; Q-12; Santa Cruz Biotechnology, USA) and Fibrillin-1 (1:100; ab53076; Abcam, UK). The specificity of the antibody against Adamts17 (Q-12) was confirmed by Western blot analysis, using HeLa cells overexpressing ADAMTS17 or EGFP. We did not confirm the specificity for the antibody against Fibrillin-1 (ab53076), because this antibody has been widely used for immunohistochemistry in previous studies^60,61^. For single immunohistochemical testing using an antibody against Adamts17, the sections were subsequently treated with Anti-Mouse Envision-Plus System-HRP (DAKO, Denmark) for 30 min. Peroxidase labeling was visualized using peroxidase substrate 3,3ʹ-diaminobenzidine and counterstaining with methyl green. For double staining against Adamts17 and Fibrillin-1, anti-mouse Alexa Fluor 488 (1:1000; Invitrogen, USA) and anti-rabbit Alexa Fluor 594 (1:1000; Invitrogen, USA) secondary antibodies were used for visualization. Finally, sections were analyzed using fluorescence microscopy (BZ-8100; KEYENCE, Japan). For both mouse and human IVD samples, IgG was used as a negative control.

### Cell culture

MG63 cells were obtained from Health Science Research Resources Bank (Tokyo, Japan) and maintained in Dulbecco’s modified Eagle’s medium containing 10% (v/v) fetal bovine serum (FBS) supplemented with penicillin and streptomycin at 37 °C in a humidified atmosphere containing 5% CO_2_^31^. MG63 cells are a human osteosarcoma cell line. We used this cell line in this study because MG63 cells were reported to produce fibrillin-1-containing fibrillar matrices^31^. For qRT-PCR, MG63 cells transfected with vectors were lysed and homogenized using TRIzol Reagent (Invitrogen, USA), and total RNA was isolated, using an RNeasy Mini Kit (Qiagen, Germany).

### Microfibril biogenesis assay and immunocytochemistry

MG63 cells were seeded at a density of 90% confluence on a 6-well dish^31^. After 24 h, FBS and antibiotics were removed, and cells were transfected using an expression vector of ADAMTS17 or empty vector. The amount of transfected vector used was 4000 ng, and transfection was conducted with 10 mL of Lipofectamine 2000 (Invitrogen, USA). On the following day, transfected cells were seeded on chamber slides and cultured at 37 °C with fresh medium supplemented with 10% FBS and antibiotics. Four days later, immunocytochemistry was conducted. After washing with phosphate-buffered saline (PBS), cells were fixed using methanol at − 20 °C for 7 min and washed with PBS thrice. After blocking with 1% bovine serum albumin (BSA) in PBS for 10 min, cells were washed thrice with PBS and then incubated with anti-fibrillin-1 monoclonal antibody (1:100; MAB1919, Merck Millipore, USA) at 4 °C overnight to detect fibrillin-1 produced by the MG63 cells. After washing thrice with PBS, anti-mouse Alexa Fluor 568 (1:500; Invitrogen, USA) was used as a secondary antibody. Nuclei were visualized using Hoechst 33342. Fluorescent images were recorded by fluorescence microscopy (BZ-8100; KEYENCE, Japan). For the quantification of the extracellular microfibrils, positive areas and signal intensities were automatically quantified using a hybrid cell count application in the BZ-X Analyzer software (KEYENCE, Japan).

### Evaluation of lumbar IVDs in patients with Marfan syndrome and idiopathic scoliosis

Consecutive patients with Marfan syndrome who underwent surgery for scoliosis in the University of Tokyo hospital from 01 April 2010 to 31 March 2020 were selected. Exclusion criteria were as follows: (1) preoperative MRI of the lumbar spine were not available and (2) the age when MRI was performed was less than 10 years old. As a result, 19 patients with Marfan syndrome were enrolled. Among these 19 patients, 16 were verified to harbor mutations in *FBN1*. In the remaining three patients, genetic testing was not performed at the patients’ or their guardians’ request; however, they were highly predicted to have *FBN1* mutations because all three cases had a family history of Marfan syndrome and were diagnosed with Marfan syndrome according to the revised Ghent nosology^62^. The preoperative MRI of the lumbar spine of these patients were compared with those of age- and sex-matched patients with idiopathic scoliosis who underwent surgery for idiopathic scoliosis in the same hospital during the same period. In total, 38 control patients with idiopathic scoliosis were selected, because two control patients with idiopathic scoliosis were allocated for each case with Marfan syndrome. The matched age between the groups was the age when MRI was performed. The degree of the degeneration of lumbar IVDs was determined according to Pfirrmann’s grading system, and Pfirmann’s grade 3 or more was defined as degeneration in this study^63^. Five lumbar IVDs from L1/2 to L5/S were evaluated for each patient. The evaluation of the IVDs was conducted by two attending spine surgeons (Y.T. and Y.O.) in a blinded manner. The final decision of the grading was made after an agreement from the two surgeons. Cobb angle of scoliosis in each patient was measured on whole spine X-ray in the standing position.

### Statistical analysis

Continuous variables were compared using a *t*-test. The chi-square test was used to compare the conditions of IVDs in patients with Marfan syndrome and idiopathic scoliosis. Statistical significance was set at P < 0.05. Statistical analyses were performed using *JMP Pro* 15 (SAS Institute Inc., Cary, NC, USA).

### Ethics approval for animal experiments

All experiments were performed according to protocols approved by the Animal Care and Use Committee of The University of Tokyo. All methods were carried out in accordance with Guidelines for Proper Conduct of Animal Experiments established by Science Council of Japan. All methods were reported in accordance with Animal Research: Reporting of In Vivo Experiments (ARRIVE) guidelines.

### Ethics approval and consent to participate

This study was performed in line with the principles of the Declaration of Helsinki. Approval was granted by the Ethics Committee of the University of Tokyo (G1326-(6), 2674-(6)). Written informed consent was obtained from all participants*.*

## Supplementary Information

Supplementary Information 1.

Supplementary Information 2.

## Data Availability

GWAS data in this study is available from Supplementary File (SI_GWAS_datafile.csv). The other datasets used and/or analyzed during the current study are available from the corresponding author on reasonable request.

## Abbreviations

AF: Annulus fibrosus
BMI: Body mass index
BSA: Bovine serum albumin
CI: Confidence interval
DSN: Disc space narrowing
ECM: Extracellular matrix
FBS: Fetal bovine serum
GWAS: Genome-wide association study
HWE: Hardy–Weinberg equilibrium
IVD: Intervertebral disc
LD: Linkage disequilibrium
LDD: Lumbar disc degeneration
MAF: Minor allele frequency
MRI: Magnetic resonance imaging
NP: Nucleus pulposus
OA: Osteoarthritis
OR: Odds ratio
PBS: Phosphate-buffered saline
SNP: Single nucleotide polymorphism
TGFβ: Transforming growth factorβ
