# Supplementary material for: A common variant rs2054564 in ADAMTS17 is associated with susceptibility to lumbar spondylosis
Source: Sci Rep. 2023 Mar 25;13:4900. doi: 10.1038/s41598-023-32155-w (PMC10039864; doi:10.1038/s41598-023-32155-w)
Supplement: Supplementary file 2 — Supplementary Information 2. [file 41598_2023_32155_MOESM2_ESM.pdf]

Supplementary information:

Title

A common variant rs2054564 in *ADAMST17* is associated with susceptibility to lumbar spondylosis.

Author list

Yuki Taniguchi*, Toru Akune, Nao Nishida, Go Omori, Kim HA, Kazuko Ueno, Taku Saito, Takeshi Oichi, Asako Koike, Akihiko Mabuchi, Hiroyuki Oka, Shigeyuki Muraki, Yasushi Oshima, Hiroshi Kawaguchi, Kozo Nakamura, Katsushi Tokunaga, Sakae Tanaka, Noriko Yoshimura

*Correspondence should be addressed to: Yuki Taniguchi, M.D., Ph.D.

Supplementary Table S1. Previously identified disease-associated SNPs and susceptibility genes associated with lumbar disc degeneration or lumbar spondylosis

Disease-associated variants	Susceptibility gene	Study design	Ethnicity	Total number of cases vs controls	Target disease	Definition of phenotype
rs1676486 [1]	*COL11A1*	Association study for a specific gene	East Asian (Japanese)	823 vs 838	LDH	LDH causing sciatic pain
rs9406328, rs17576 [2]	*THBS2, MMP9*	Association study for a specific gene	East Asian (Japanese)	847 vs 896	LDH	LDH causing sciatic pain
rs143383 [3]<br>(in only females)	*GDF5*	Association study for a specific gene	Northern European	478 vs 4,781	LDD	DSN and osteophyte formation
rs16924573 [4]	*SKT (KIA1277)*	Association study for a specific gene	East Asian (Japanese)	862 vs 896	LDH	LDH causing sciatic pain
ASPN D-14 allele [5]	*ASPN*	Association study for a specific gene	East Asian (Chinese)<br>East Asian (Japanese)	527 vs 528<br>745 vs 608	LDD<br>LDH	LDD assessed by magnetic resonance imaging<br>LDH causing sciatic pain
rs2073711 [6]	*CILP*	Association study for specific genes	East Asian (Japanese)	467 vs 654	LDH	LDH causing sciatic pain
rs4148941 [7]	*CHST3*	GWAS	East Asian (Chinese)<br>East Asian (Japanese)<br>Northern European (Finnish)	4,043 vs 28,599	LDH<br>LDD<br>LDH	LDH causing sciatic pain<br>LDD assessed by magnetic resonance imaging<br>LDH causing sciatic pain
rs926849 [8]	*PARK2*	GWAS	Northern European	4,683 subjects in total	LDD	DSN and osteophyte formation
(rs11224760 [9])<br>Not statistically significant		GWAS	East Asian (Koreans)	1,029 vs 395	LS	Kellgren-Lawrence grade ≥ 2
rs12190551 [10]<br>rs12499841 [10]	*BMP6, NIPAL1, CNGA1*	GWAS	Mostly Western Europeans	4,434 vs 12,522	LS	ICD diagnosis codes

DSN, Disc space narrowing; GWAS, genome-wide association study; Lumbar disc herniation; LDH, Lumbar disc herniation; LS, Lumbar spondylosis

<Reference for Supplementary Table S1>

1. Mio F, et al. A functional polymorphism in COL11A1, which encodes the alpha 1 chain of type XI collagen, is associated with susceptibility to lumbar disc herniation. *Am J Hum Genet*. **81**, 1271–1277 (2007).
2. Hirose Y, et al. A functional polymorphism in THBS2 that affects alternative splicing and MMP binding is associated with lumbar-disc herniation. *Am J Hum Genet*. **82**, 1122–1129 (2008).
3. Williams FM, et al. GDF5 single-nucleotide polymorphism rs143383 is associated with lumbar disc degeneration in northern European women. *Arthritis Rheum*. **63**, 708–712 (2011).
4. Karasugi T, et al. Association of the tag SNPs in the human SKT gene (KIAA1217) with lumbar disc herniation. *J Bone Miner Res*. **24**, 1537–1543 (2009).
5. Song YQ, et al. Association of the asporin D14 allele with lumbar-disc degeneration in Asians. *Am J Hum Genet*. **82**, 744–747 (2008).
6. Seki S, et al. A functional SNP in CILP, encoding cartilage intermediate layer protein, is associated with susceptibility to lumbar disc disease. *Nat Genet*. **37**, 607–612 (2005).
7. Song YQ, et al. Lumbar disc degeneration is linked to a carbohydrate sulfotransferase 3 variant. *J Clin Invest*. **123**, 4909–4917 (2013).
8. Williams FM, et al. Novel genetic variants associated with lumbar disc degeneration in northern Europeans: a meta-analysis of 4600 subjects. *Ann Rheum Dis*. **72**, 1141–1148 (2013).
9. Kim HA, Heo SG, Park JW, Jung YO. Novel genetic variants associated with lumbar spondylosis in Koreans: A genome-wide association study. *J Korean Neurosurg* Soc. **61**, 66–74 (2018). PMID: 29354237, PMCID: PMC5769851
10. Zhang Y, et al. Genome-wide association analysis across 16,956 patients identifies a novel genetic association between BMP6, NIPAL1, CNGA1 and spondylosis. *Spine (Phila Pa 1976*). **46**, E625–E631 (2021).

Supplementary Table S2. Results of the original GWAS, imputed whole-genome sequence-based GWAS, and meta-analysis in four cohorts for 65 selected SNPs.

					Original GWAS	GWAS based on imputed genotypes		Meta-analysis of four cohorts	
SNP ID	Chromosome	MAF	Risk	Ref.	P-value	SNPs with the smallest P-value in ±500 kb	P-value	Odds ratio (95% CI)	P value
rs9442385	1	0.375	G	T	0.00002168			1.04 (0.87, 1.24)	0.7314
rs1188356	1	0.197	G	T	0.0002887			1.21 (0.97, 1.52)	0.1087
rs1317587	1	0.37	A	T	0.00006503			0.94 (0.78, 1.12)	0.5122
rs12077047	1	0.386	T	A	0.0007334			0.97 (0.81, 1.16)	0.7515
rs680341	1	0.244	G	T	0.0001486			0.97 (0.79, 1.19)	0.7962
rs4993338	2	0.447	C	G	0.000483			0.95 (0.8, 1.13)	0.5511
rs6727165	2	0.464	C	G	0.00002135			1.03 (0.87, 1.22)	0.7327
rs34165704	3	0.158	A	G	0.0000179			1.02 (0.8, 1.3)	0.8249
rs12491849	3	0.261	T	C	0.0009404			1.07 (0.88, 1.3)	0.4424
rs9862203	3	0.385	A	G	0.0001193			1.06 (0.88, 1.26)	0.5359
rs936359	4	0.384	T	G	0.00006465			0.98 (0.82, 1.18)	0.8742
rs2946386	4	0.422	C	G	0.00003222			1.12 (0.94, 1.33)	0.2267
rs2331856	4	0.143	G	A	0.0007916			1.21 (0.94, 1.57)	0.1871
rs10037203	5	0.188	C	T	0.0006901			1.19 (0.94, 1.49)	0.1628
rs2925178	5	0.31	C	A	0.0002015			0.93 (0.77, 1.12)	0.4349
rs12516971	5	0.391	C	T	0.0007539			1.03 (0.86, 1.24)	0.8015
rs7732882	5	0.316	A	G	0.00001192			1.16 (0.96, 1.4)	0.1426
rs252062	5	0.255	A	T	0.0007985			1.04 (0.85, 1.27)	0.662
rs952188	6	0.331	G	A	0.0002627			0.93 (0.77, 1.12)	0.4463
rs6569814	6	0.288	A	C	0.0006611	rs6936426	0.0003558	0.96 (0.79, 1.16)	0.6728
rs1931867	6	0.313	G	A	0.0004978			1.14 (0.95, 1.37)	0.1908
rs719114	7	0.417	C	T	0.00003859			0.99 (0.83, 1.18)	0.9253
rs7844884	8	0.244	T	A	0.0002981			1.07 (0.87, 1.3)	0.5314
rs13268751	8	0.459	T	C	0.000000349	rs17239618, rs4397389	0.0000003136	0.97 (0.82, 1.16)	0.7522
rs7009797	8	0.454	C	A	0.000006712			1.01 (0.85, 1.2)	0.9289
rs10114878	9	0.439	C	T	0.0008934			1.15 (0.97, 1.37)	0.1139
rs10963564	9	0.248	A	G	0.0003075			1.18 (0.96, 1.44)	0.1022
rs11793406	9	0.303	G	A	0.0001334	rs10965400	0.00006911	1.01 (0.84, 1.22)	0.9084
rs7854748	9	0.42	G	A	0.0001779			1.17 (0.98, 1.39)	0.07956
rs10760307	9	0.493	G	C	0.0002493			0.95 (0.8, 1.13)	0.5481
rs876663	9	0.405	T	C	0.00003192			1.17 (0.98, 1.39)	0.09196
rs650599	9	0.318	G	T	0.00003554			0.89 (0.73, 1.07)	0.2311

rs2279211	10	0.367	G	T	0.0008476	rs11251540	0.0002124	1.32 (1.09, 1.61)	0.005181
rs10905917	10	0.24	C	G	0.00007974			0.95 (0.77, 1.16)	0.6426
rs10765219	11	0.123	T	G	0.0002118	rs11018666, rs11018667, rs10830293,	0.0002055	0.93 (0.71, 1.24)	0.802
rs574075	11	0.366	T	C	0.0009382			1 (0.84, 1.2)	0.9634
rs10878396	12	0.299	C	T	0.0006719			1.08 (0.89, 1.3)	0.4229
rs2439733	12	0.35	A	G	0.0005675			1.18 (0.99, 1.42)	0.05933
rs7971523	12	0.394	C	G	0.00007315			1.13 (0.94, 1.34)	0.1863
rs259734	13	0.221	G	T	0.0001015			1.1 (0.9, 1.36)	0.3382
rs1462031	13	0.206	C	T	0.0003807			1.1 (0.88, 1.37)	0.435
rs7989210	13	0.4	C	T	0.00001044			1.01 (0.85, 1.2)	0.9355
rs11157273	14	0.465	A	T	0.0000505			1.08 (0.91, 1.29)	0.379
rs10134030	14	0.349	A	G	0.00005705			1 (0.82, 1.22)	0.9924
rs4902492	14	0.129	C	T	0.0004983			1.03 (0.79, 1.34)	0.7935
rs7178180	15	0.183	T	C	0.0002969			0.98 (0.78, 1.23)	0.9705
rs11073977	15	0.436	A	C	0.0005862			0.93 (0.78, 1.11)	0.4078
rs1383543	15	0.445	G	A	0.0004628			1.08 (0.9, 1.28)	0.4112
rs2054564	***15***	***0.277***	***C***	***T***	***0.0002767***			***1.87 (1.52, 2.28)***	***3.05E-09****
rs11076193	16	0.332	C	T	0.0004255			1.08 (0.9, 1.3)	0.4483
rs424821	16	0.387	C	G	0.0003269			1.05 (0.88, 1.25)	0.5858
rs1622321	16	0.402	C	T	0.0003904			1.02 (0.84, 1.23)	0.8454
rs9303064	17	0.366	C	T	0.00005733			0.97 (0.81, 1.16)	0.756
rs806917	17	0.354	A	G	0.0002024			1.04 (0.87, 1.25)	0.6468
rs11652711	17	0.173	C	T	0.0002998			1.11 (0.88, 1.39)	0.3357
rs4789855	17	0.427	T	C	0.0004879			1 (0.84, 1.19)	0.9673
rs3786371	18	0.272	G	A	0.0003239			1.12 (0.93, 1.36)	0.2276
rs9304345	18	0.492	A	T	0.0003006			1 (0.84, 1.19)	0.9879
rs4804314	19	0.327	C	T	0.0008762	rs10416597	0.0008553	1.26 (1.05, 1.51)	0.0102
rs2419722	19	0.251	G	A	0.0005442			0.96 (0.78, 1.18)	0.7341
rs565833	20	0.222	T	C	0.000174			1.16 (0.94, 1.43)	0.1263
rs7353574	20	0.373	A	G	0.00005352			0.97 (0.81, 1.16)	0.826
rs4920086	21	0.479	T	G	0.0001595			1.07 (0.9, 1.27)	0.4712
rs5764957	22	0.301	A	G	0.0002163			0.99 (0.82, 1.19)	0.9478
rs11704217	22	0.288	A	G	0.0005532			1.08 (0.89, 1.3)	0.4272

MAF, minor allele frequency; Ref., reference; GWAS, genome-wide association study; CI, confidence interval; SNP, single nucleotide polymorphism

Only one SNP rs2054564 reached a genome-wide significance threshold in the meta-analysis of four cohorts, which included Tokyo-2, Wakayama-1, Wakayama-2, and Niigata cohort.

Supplementary Table S3. Databases used for genotype imputation.

Database used for genotype imputation	Reference	Number of subjects	Availability of datasets
International 1000 Genomes	[1]	2,493	Available by download from the public databases.
Human Genome Diversity Project	[2]	820	Available by download from the public databases.
Simons Genome Diversity Project	[3]	241	Available by download from the public databases.
Korean Personal Genome Diversity Project	[4]	90	Available by download from the public databases.
Biobank Japan		1,026	Controlled access data from NBDC human database (JGAS000114).
Tokyo Healthy Control Project	[5]	418	Available by download from the public databases.

<Reference for Supplementary Table S3>

1. Byrska-Bishop M, et al. High-coverage whole-genome sequencing of the expanded 1000 Genomes Project cohort including 602 trios. *Cell*. **185**, 3426-3440.e19 (2022). PMID: 36055201; PMCID: PMC9439720.
2. Bergström A, et al. Insights into human genetic variation and population history from 929 diverse genomes. *Science*. **367**, eaay5012 (2020). PMID: 32193295, PMCID: PMC7115999
3. Mallick S, et al. The Simons Genome Diversity Project: 300 genomes from 142 diverse populations. *Nature*. **538**, 201–206 (2016). Epub 2016 Sep 21. PMID: 27654912, PMCID: PMC5161557
4. Kim J, et al. KoVariome: Korean National Standard Reference Variome database of whole genomes with comprehensive SNV, indel, CNV, and SV analyses. S*ci Rep.* **8**, 5677 (2018). PMID: 29618732, PMCID: PMC5885007
5. Nishida N, et al. Evaluating the performance of Affymetrix SNP Array 6.0 platform with 400 Japanese individuals. *BMC Genomics*. **9**, 431 (2008).

Supplementary Table S4. Primers used for qRT-PCR

Gene	Species	Reference sequence	Product size	Annealing temperature for qPCR	Primer sequence		Melting temperature (Tm) of primer
Col1a1	Mouse	NM_007742.4	170 bp	60˚C	F	ACGTCCTGGTGAAGTTGGTC	58.8˚C
					R	CAGGGAAGCCTCTTTCTCCT	58.3˚C
Col2a1	Mouse	NM_031163.4	239 bp	60˚C	F	GCCAAGACCTGAAACTCTGC	58.3˚C
					R	GCCATAGCTGAAGTGGAAGC	58.2˚C
Acan	Mouse	NM_001361500.1	165 bp	60˚C	F	CCAAACCAGCCTGACAACTT	57.0˚C
					R	TCTAGCATGCTCCACCACTG	57.1˚C
Skt	Mouse	NM_178059.5	208 bp	60˚C	F	CCATGTCCGAGGGAGAAGTA	57.6˚C
					R	GGAAAGGCACTGACGAAGAG	57.6˚C
Cd24	Mouse	NM_009846.2	163 bp	60˚C	F	CTTCTGGCACTGCTCCTACC	59.8˚C
					R	GAGAGAGAGCCAGGAGACCA	60.2˚C
Adamts17	Mouse	NM_001033877.4	244 bp	60˚C	F	ACCATGATGACGACCACTCA	57.0˚C
					R	AGAATCTGGCACTGCTCGTT	58.0˚C
Fbn1	Mouse	NM_007993.2	217 bp	60˚C	F	TGCCACAGTCCATAACCAAA	55.5˚C
					R	GGAGGATAGCCAGGAGGAAC	59.3˚C
β-actin	Mouse	NM_007393.5	125 bp	60˚C	F	AGATGTGGATCAGCAAGCAG	56.3˚C
					R	GCGCAAGTTAGGTTTTGTCA	55.1˚C
ADAMTS17	Human	NM_139057.4	152 bp	60˚C	F	CTAATCCAGCCCCTCAACAA	59.0˚C
					R	CACGTCGGCTTCTTCTTTTC	58.6˚C
GAPDH	Human	NM_001289745.3	226 bp	60˚C	F	GAAGGTGAAGGTCGGAGTCA	60.7˚C
					R	GAAGATGGTGATGGGATTTC	55.2˚C

bp; base pairs; qRT-PCR, quantitative real-time polymerase chain reaction.

Supplementary Figure S1. Manhattan plot of a genome-wide association study conducted as a screening. Eighty-three Japanese subjects with severe lumbar spondylosis from Tokyo-1 cohort and 182 healthy control subjects were compared. P-values were calculated by chi-squared test for allele frequencies.

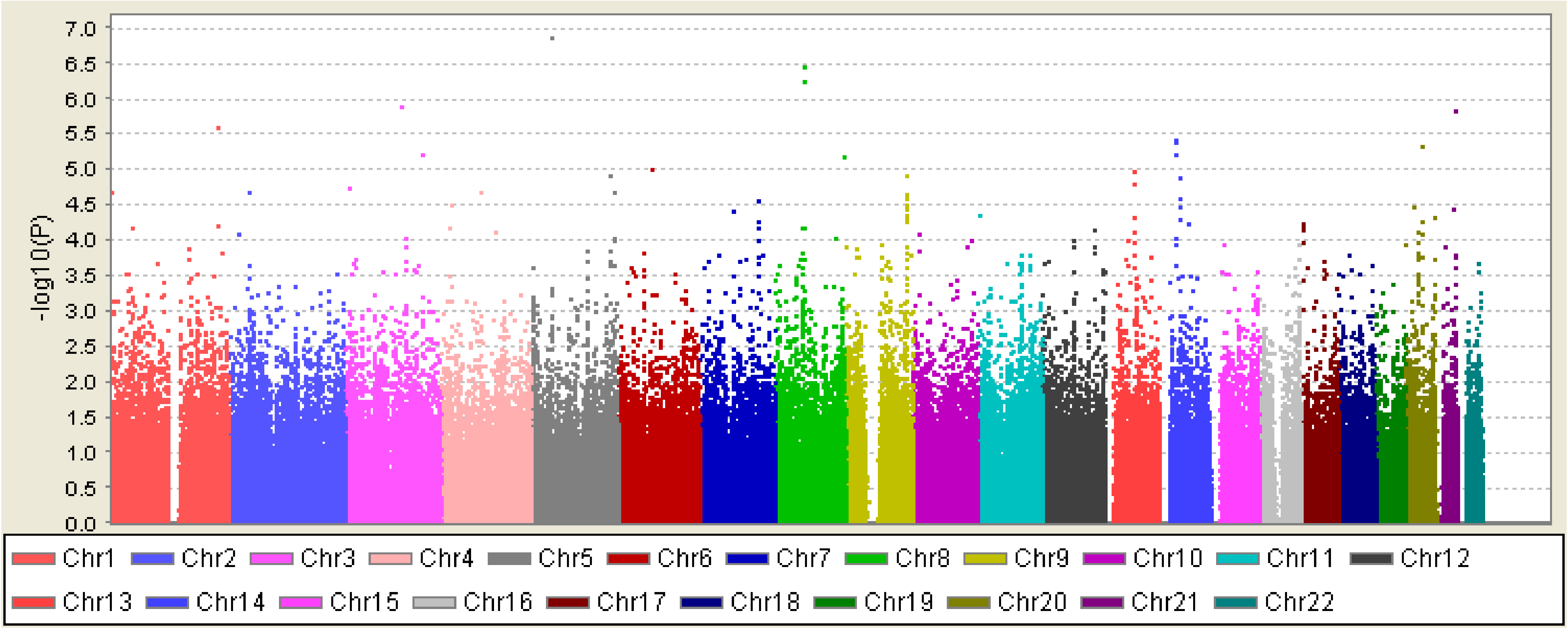

Supplementary Figure S2. Principal component analysis of 83 cases from Tokyo-1 cohort and 182 healthy controls was conducted to check the genetic background in the studied samples together with the HapMap samples.

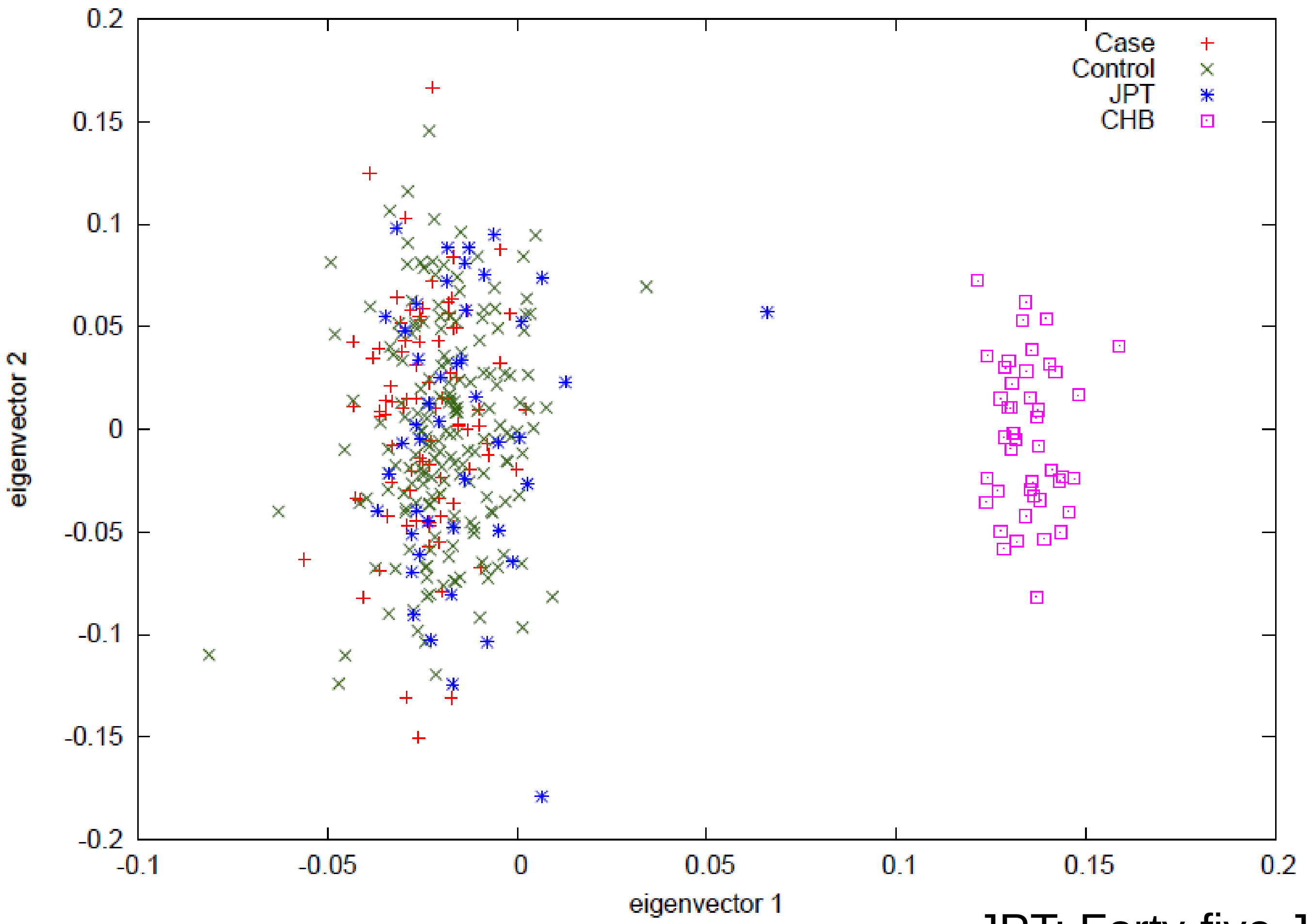

JPT: Forty-five Japanese individuals from Hap Map database [1]
CHB: Forty-five Han Chinese individuals from Hap Map database [1]

[1] International HapMap Consortium. A haplotype map of the human genome. *Nature*. **437**: 1299-1320 (2005).
